# Draft Genome Sequence of Enterobacter mori Strain NSE2, Isolated from the Rhizosphere of a *Sedum* sp.

**DOI:** 10.1128/MRA.00487-21

**Published:** 2021-08-26

**Authors:** Charlotte J. François, Steven Batinovic, Steve Petrovski, Anthony R. Gendall

**Affiliations:** a Department of Animal, Plant and Soil Sciences, La Trobe University, Bundoora, Victoria, Australia; b Department of Physiology, Anatomy and Microbiology, La Trobe University, Bundoora, Victoria, Australia; University of Southern California

## Abstract

Enterobacter mori is an important plant pathogen. Here, we report the draft genome sequence of the plant-associated strain Enterobacter mori NSE2, which was found to harbor genes for promotive and pathogenic interactions with plants.

## ANNOUNCEMENT

Enterobacter bacteria are broadly spread through a range of habitats. They are found in diverse environments like water, soil, and the systems of animals, humans, and plants (1), where they have a range of effects on their hosts. Enterobacter mori causes bacterial wilt in economically important mulberry trees (Morus alba), which are the primary food source of silkworms (Bombyx mori), whose cocoons are used to make silk (2). Enterobacter mori strain NSE2 is a plant growth-promoting bacterium isolated from the rhizosphere of a *Sedum* sp. grown in cadmium-contaminated soil in a greenhouse pot experiment (3).

*E. mori* NSE2 was grown for 16 h at 27°C in Luria-Bertani liquid medium before genomic DNA was extracted using the Bioline isolate II genomic DNA kit and sequencing libraries created using the Nextera XT DNA library prep kit (Illumina) with 1 ng of input DNA, per the manufacturer’s guidelines. The concentrations and quality of the genomic DNA were determined using an Invitrogen Qubit 3.0 fluorometer. The libraries were sequenced using a MiSeq v.3 reagent kit (600 cycles), which produced 895,345 reads consisting of 300-bp paired-end reads on the Illumina MiSeq platform. The reads were trimmed to a Phred threshold quality score of 20 using Trimmomatic v.0.36.6 (4) and assembled using SPAdes v.3.12 (5), both through Galaxy Australia (6). The final length of the assembled genome sequence of *E. mori* NSE2 was 5,519,809 bp, with a G+C content of 54.86%. There were 35 contigs with an *N*_50_ value of 251,350 bp. The final genome coverage was 35×. Default parameters were used for all software unless stated otherwise.

The assembled genome was annotated using the Prokaryotic Genome Annotation Pipeline (PGAP) v.4.10 of the National Center for Biotechnology Information (NCBI) (7). Average nucleotide identity (ANI) analysis using JSpeciesWS v.3.8.2 revealed a high sequence similarity (96.41%) to the genome of *E. mori* LMG 25706 (8). In total, 5,245 genes and 98 RNA genes were identified from the annotation. There were 5,147 coding sequences (CDS) present, and of the RNA genes, there were 7 5S rRNAs, 2 16S rRNAs, 2 32S rRNAs, and 79 tRNAs.

Genes involved in beneficial interactions with plants were identified in the annotated genome sequence of *E. mori* NSE2. The *fliC* (flagellin) gene, which encodes the filament subunit of flagella, is a strong immunomodulator that triggers induced systemic resistance (ISR) in plants (9). The activation of ISR primes the host plant immune system in anticipation of future attack by pathogens (10). The genome also contains genes encoding the genus-specific siderophores bacterioferritin (*fepB*, *fepC*, and *fepD*) and enterobactin (*entC*, *entD*, *entE*, and *entF*), which may be responsible for improving iron availability in limiting environments (11). Subsequent analysis could contribute to a better understanding of the promotive and pathogenic traits of this strain on plant hosts.

### Data availability.

The *E. mori* NSE2 whole-genome sequencing project has been deposited at GenBank under accession number JAAAJY000000000, BioProject accession number PRJNA600758, and BioSample accession number SAMN13831071. The raw sequences were deposited at the Sequence Read Archive under accession number SRR14460588.
